# Adverse events following the first, second and third doses of a COVID-19 vaccine in hemodialysis patients

**DOI:** 10.1080/0886022X.2023.2172432

**Published:** 2023-01-30

**Authors:** Mei-Fen Pai, Kuei-Ting Tung, Shih-Ping Hsu, Yu-Sen Peng, Wan-Yu Lin, Ju-Yeh Yang, Hon-Yen Wu, Yen-Ling Chiu, Kai-Hsiang Shu, Wan-Chuan Tsai

**Affiliations:** a Division of Nephrology, Department of Internal Medicine, Far Eastern Memorial Hospital, New Taipei City, Taiwan; b Department of Internal Medicine, National Taiwan University Hospital and College of Medicine, Taipei City, Taiwan; c School of Life Science, National Taiwan Normal University, Taipei City, Taiwan; d Department of Applied Cosmetology, Lee-Ming Institute of Technology, New Taipei City, Taiwan; e Department of Electrical Engineering, Yuan Ze University, Taoyuan City, Taiwan; f Institute of Epidemiology and Preventive Medicine, College of Public Health, National Taiwan University, Taipei City, Taiwan; g Center for General Education, Lee-Ming Institute of Technology, New Taipei City, Taiwan; h Faculty of Medicine, School of Medicine, National Yang-Ming University, Taipei City, Taiwan; i Graduate Program in Biomedical Informatics, Yuan Ze University, Taoyuan City, Taiwan

**Keywords:** Hemodialysis, adverse event, vaccination, COVID-19, ChAdOx1, mRNA-1273

## Abstract

**Background:**

This study aimed to identify adverse events following the first three doses of COVID-19 vaccines in hemodialysis (HD) patients. Risk factors associated with postvaccination adverse events were explored.

**Methods:**

Postvaccination adverse events in 438 HD patients who received 3 doses of COVID-19 vaccines were prospectively assessed. The adverse events among three doses were compared using generalized linear mixed models. Factors associated with adverse events were assessed with multivariate analyses.

**Results:**

The vast majority of participants received Oxford/AstraZeneca ChAdOx1 as their first two doses and Moderna mRNA-1273 as their third dose. Overall, 79%, 50% and 84% of the participants experienced at least one adverse event after their first, second, and third doses, respectively. These adverse events were mostly minor, short-lived and less than 5% reported daily activities being affected. Compared with the first dose, the second dose caused a lower rate of adverse events. Compared with the first dose, the third dose elicited a higher rate of injection site reactions and a lower rate of systemic reactions. Multivariate analyses showed that every 10-year increase of age (odds ratio 0.67, 95% confidence intervals 0.57-0.79) was associated with decreased risk of adverse events, while female sex (2.82, 1.90-4.18) and arteriovenous fistula (1.73, 1.05–2.84) were associated with increased risk of adverse events. Compared with Oxford/AstraZeneca ChAdOx1, Moderna mRNA-1273 was associated with an increased risk of injection site reactions.

**Conclusions:**

COVID-19 vaccination was well tolerated in HD patients. Age, sex, dialysis vascular access and vaccine types were associated with postvaccination adverse events.

## Introduction

Coronavirus disease 2019 (COVID-19) has spread across the world, resulting in over 603 million cases and over 6.4 million deaths globally [1]. From the beginning of the pandemic until early September 2022, Taiwan has reported more than 5.4 million confirmed cases of COVID-19, resulting in more than 10,000 deaths [2]. Global coverage of vaccination has been accepted as being essential for effective control of the COVID-19 pandemic. In Taiwan, the COVID-19 vaccine rollout began in March 2021. By 3 September 2022, the majority of inhabitants in Taiwan had received COVID-19 vaccines (the coverage of the first, second and third doses was 92.9%, 86.8%, and 72.4%, respectively) [2].

Patients on chronic hemodialysis (HD) are particularly vulnerable, and effective vaccination coverage is essential because of their susceptibility to COVID-19-related morbidity and mortality. Advanced chronic kidney disease was reported to be associated with a 9-fold increase of COVID-19-related hospitalization [3] and a 3-fold increase of COVID-19 attributable death [4]. The 28-day COVID-19-attributable mortality was reported to be around 20 percent in patients on maintenance dialysis [5,6]. Furthermore, self-quarantine is almost impossible for HD patients since they typically receive in-center dialysis therapy three times a week. An increasing number of studies are addressing the efficacy of COVID-19 vaccines in dialysis populations with valid immunological responses and immunosuppressive therapy was reported to be associated with the risk of being a nonresponder [7–9]. However, our recent study showed that the seropositivity rate in HD patients was low, and most had low antibody titers after the first dose of the Oxford/AstraZeneca ChAdOx1 nCoV-19, (ChAdOx1) vaccine [10]. The lack of information regarding the safety of these vaccines in this specific population has been a major cause of vaccine hesitancy among both patients and physicians [11]. Clarification of the adverse events of COVID-19 vaccines will help to ease the anxiety associated with vaccination and to increase these patients’ willingness to receive further vaccines. Therefore, the objective of this study was to survey the frequency, duration, spectrum, and risk factors of adverse events following the first, second, and third doses of COVID vaccines in patients on maintenance HD.

## Materials and methods

### Study population

This prospective observational study was conducted in the HD unit of Far Eastern Memorial Hospital, a tertiary medical center located in New Taipei City. Subjects were asked to participate if they met the following inclusion criteria: (1) were aged older than 20 years; (2) had end-stage kidney disease (ESKD) and were undergoing HD; and (3) were vaccinated or willing to receive the vaccination. All participants provided written informed consent.

### Assessment of adverse events following vaccination

The frequency and duration of adverse events following the first, second, and third doses of COVID-19 vaccines were recorded using a Google Form, an interactive web-based questionnaire. For each of the participants, the questionnaire was completed with the assistance of dialysis staff during routine hemodialysis therapy. In addition to general demographic, comorbidity, and vaccine-related data, the questionnaire involved the following 8 major domains dedicated to the safety profile of the COVID-19 vaccine: (1) any injection site reaction, including redness, itching, pain, ecchymosis, and nodules; (2) fever, classified into fever (temperature ≥ 38 °C), mild fever (temperature 37.5 ∼ 37.9 °C), and fever with unknown temperature; (3) any bodily pain, which included headache, myalgia, arthralgia, and chest pain; (4) gastrointestinal reactions, such as poor appetite, nausea, vomiting, and diarrhea; (5) skin reactions at noninjection sites, including rash, redness of mucosa and itching; (6) other adverse reactions, such as chills, malaise, sleepiness, shortness of breath, chest tightness, and dizziness; (7) anaphylactic reactions; and (8) reactions requiring medications, which included oral and intravenous medications. All the participants were questioned about these adverse events on day 7 after each dose of vaccination, with day 0 defined as the day of vaccination. To assess the severity of adverse events, participants were also asked if their daily activity was affected and/or whether they sought medical attention (e.g., visited clinics). To document serious adverse events such as vaccine-induced immune thrombotic thrombocytopenia and myocarditis, the study participants’ electronic medical records were followed for 3 months after vaccination.

### Clinical data collection

All clinical data were obtained at enrollment, including age, sex, body mass index, diabetes, hypertension, vaccine type, dialysis access type, Kt/V (Daugirdas), albumin, hemoglobin, ferritin, and intact parathyroid hormone. The frequency and duration of adverse events were recorded regarding the duration at 7 days following the first, second, and third doses of COVID-19 vaccines.

### Statistical analysis

Continuous variables are expressed as the means (± SDs) or medians (1^st^ and 3^rd^ quartiles), and categorical variables are expressed as counts and percentages. We used generalized linear mixed models (GLMMs) that incorporated three repeated measurements of adverse event data to compare the frequency and duration of adverse events following COVID-19 vaccination among the three doses. We specified binomial distribution in the GLMMs. The response variable was one of the adverse events (yes vs. no). Focusing on three major adverse events (any adverse event, any injection site reaction, and any systemic reaction), we also explored factors associated with these adverse events following COVID-19 vaccination after the three doses using multivariate analyses. The covariates of interest included age, sex, body mass index, diabetes, hypertension, vaccine type, dialysis access type, Kt/V (Daugirdas), albumin, hemoglobin, ferritin, and intact parathyroid hormone. A two-sided *p* value of less than 0.05 indicated statistical significance. All analyses were performed with SAS version 9.4 software (SAS Institute).

## Results

### Characteristics of the study participants

Table 1 presents the characteristics of the study participants. In total, 438 participants who received 3 doses of COVID-19 vaccines were enrolled in this study. The mean age was 64 ± 12 years. Women accounted for 39% of the study population. The mean vintage was 6.0 ± 5.9 years. Half of the participants had diabetes, and 59% had hypertension. Nine participants (2%) were taking immunosuppressants during the study period. The median (1st, 3rd quartiles) time intervals between the first two doses of vaccines were 98 days (92, 100) and the interval between the second and the third dose was 98 days (98, 100).

**Table 1.** Characteristics of the study participants.

Characteristics	*n* = 438
Age (years)	64 ± 12
Female, *n (*%)	170 (39)
Vintage (years)	6.0 ± 5.9
Body height (cm)	162 ± 8
Body weight (kg)	63 ± 13
Body mass index (kg/m^2^)	24 ± 4
Diabetes mellitus, *n* (%)	202 (50)
Hypertension, *n* (%)	242 (59)
Immunosuppressant, *n* (%)	9 (2)
Dialysis access, *n*	438
Arteriovenous fistula, *n* (%)	348 (79)
Arteriovenous graft, *n* (%)	60 (14)
Permcath, *n* (%)	30 (7)
First vaccine type, *n*	438
ChAdOx1, *n* (%)	434 (99)
mRNA-1273, *n* (%)	4 (1)
Second vaccine type*, n*	408
ChAdOx1, *n* (%)	402 (99)
mRNA-1273, *n* (%)	6 (1)
Prime-boost regimen, *n*	408
Homologous (ChAdOx1/ChAdOx1)	402 (99)
Homologous (mRNA-1273/mRNA-1273)	2 (1)
Heterologous (ChAdOx1/mRNA-1273)	4 (1)
Third vaccine type, *n*	367
mRNA-1273, *n* (%)	356 (97)
BNT162b2, *n* (%)	6 (2)
MVC-COV1901, *n* (%)	4 (1)
ChAdOx1, *n* (%)	1 (0.3)
Kt/V (Daugirdas)	1.52 ± 0.21
Albumin (g/dL)	3.9 ± 0.4
Hemoglobin (g/dL)	11.3 ± 1.4
Ferritin (ng/mL)	371 (240, 522)
Intact parathyroid hormone (pg/mL)	192 (84, 436)

Abbreviations. ChAdOx1: Oxford/AstraZeneca ChAdOx1 nCoV-19; mRNA-1273: Moderna mRNA-1273 SARS-CoV-2; BNT162b2: BioNTech/Pfizer BNT162b2; MVC-COV1901: Medigen MVC-COV1901.

The vast majority (99%) of the participants received a homologous prime-boost regimen with ChAdOx1 vaccines. The Moderna mRNA-1273 severe acute respiratory syndrome coronavirus 2 (SARS-CoV-2) (mRNA-1273) vaccine accounted for 1% of the first dose and the second dose received by the study population. The vast majority (97%) of the participants received the mRNA-1273 vaccine as their third dose. Two percent, 1%, and 0.3% received the BioNTech/Pfizer BNT162b2 COVID-19 (BNT162b2), Medigen MVC-COV1901, and ChAdOx1 vaccines as their third dose, respectively.

### Frequency and duration of adverse effects among the study participants

**Figure 1.**
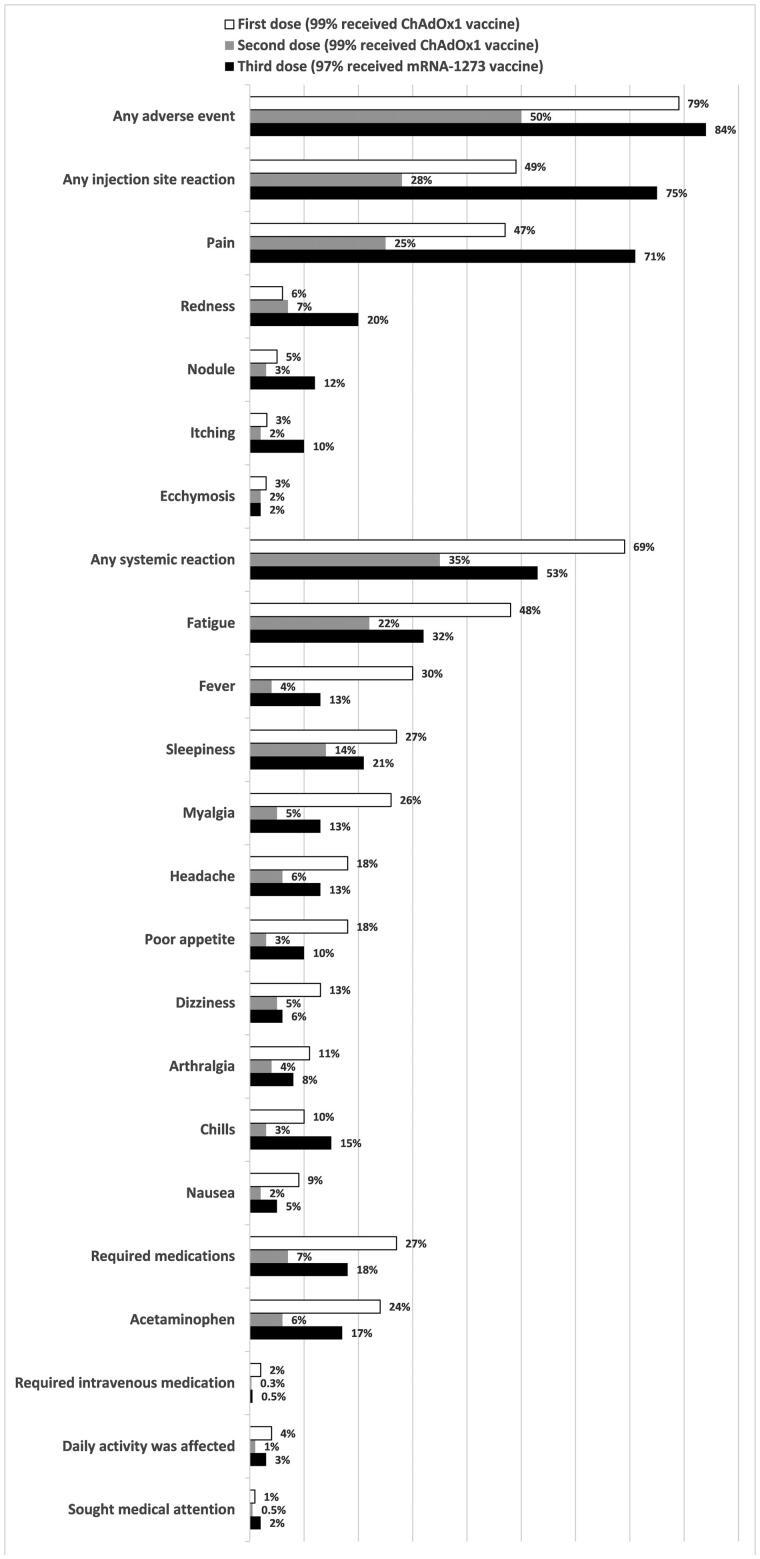
The frequency of reported adverse events within 7 days of the first, second, and third doses of a COVID-19 vaccine among the study participants. Notably, 99% of the participants received the ChAdOx1 vaccine as their first and second doses, and the mRNA-1273 vaccine accounted for 97% of their third doses. The adverse events are presented in descending order of the first COVID-19 vaccine.

Figure 1 shows the frequency of reported adverse events within 7 days of the first, second, and third doses of COVID-19 vaccination among the study participants. Overall, 79%, 50%, and 84% of the HD patients experienced at least one adverse event following the first, second, and third doses of COVID vaccines, respectively. Nearly half of HD patients reported fatigue (48%) and injection site pain (47%), followed by fever (30%), after the first dose. Following the second dose, the most common adverse events were injection site pain (25%), fatigue (22%), and sleepiness (14%). More than 70 percent (71%) of HD patients experienced injection site pain, followed by fatigue (32%) and sleepiness (21%), after the third dose.

Despite the high frequency of various postvaccination reactions, only a small number of HD patients reported that their daily activity was affected (4%, 1%, and 3% after the first, second, and third doses, respectively). Moreover, very few HD patients sought medical attention (1%, 0.5%, and 2% after the first, second, and third doses, respectively), and only a small portion of the HD patients required medication to treat postvaccination discomfort; among them, the most commonly prescribed medication was acetaminophen. No vaccine-induced immune thrombotic thrombocytopenia nor myocarditis was reported during the study period.

### Comparison of the frequency and duration of adverse events among the three doses

Table 2 describes the comparison of the frequency of adverse events following COVID-19 vaccination among the three doses. Notably, 99% of the participants received the ChAdOx1 vaccine as their first and second doses, and the mRNA-1273 vaccine accounted for 97% of their third doses.

**Table 2.** Comparison of the frequency of adverse events following COVID-19 vaccination among the three doses.

Type of reaction	First dose (*n* = 431)	Second dose (*n* = 408)	Third dose (*n* = 367)	Second vs. first		Third vs. first		Third vs. second	
	*n* (%)	*n* (%)	*n* (%)	Odds ratio (95% CI)	*p* value	Odds ratio (95% CI)	*p* value	Odds ratio (95% CI)	*p* value
Any adverse event	340 (79)	202 (50)	308 (84)	0.25 (0.18–0.34)	<0.001	1.42 (0.98–2.05)	0.07	5.77 (4.07–8.18)	<0.001
Any injection site reaction	212 (49)	113 (28)	276 (75)	0.38 (0.28–0.51)	<0.001	3.26 (2.39–4.45)	<0.001	8.63 (6.20–12.00)	<0.001
Redness	26 (6)	29 (7)	74 (20)	1.19 (0.69–2.07)	0.53	3.98 (2.47–6.40)	<0.001	3.33 (2.11–5.27)	<0.001
Itching	15 (3)	8 (2)	35 (10)	0.55 (0.23–1.32)	0.18	2.93 (1.57–5.47)	0.001	5.29 (2.41–11.59)	<0.001
Pain	204 (47)	101 (25)	259 (71)	0.35 (0.26–0.47)	<0.001	2.73 (2.02–3.69)	<0.001	7.79 (5.63–10.78)	<0.001
Ecchymosis	12 (3)	8 (2)	8 (2)	0.70 (0.28–1.73)	0.44	0.78 (0.31–1.93)	0.59	1.11 (0.41–3.00)	0.83
Nodules	20 (5)	13 (3)	44 (12)	0.68 (0.33–1.38)	0.28	2.82 (1.63–4.89)	<0.001	4.16 (2.20–7.89)	<0.001
Any systemic reaction	297 (69)	142 (35)	195 (53)	0.22 (0.17–0.30)	<0.001	0.50 (0.37–0.68)	<0.001	2.23 (1.66–3.01)	<0.001
Fever	131 (30)	17 (4)	46 (13)	0.09 (0.05–0.16)	<0.001	0.31 (0.21–0.46)	<0.001	3.31 (1.86–5.90)	<0.001
Fever (temperature ≥ 38 °C)	46 (11)	6 (1)	24 (7)	0.12 (0.05–0.29)	<0.001	0.58 (0.34–0.98)	0.04	4.80 (1.92–12.00)	<0.001
Mild fever (temperature 37.5 ∼ 37.9 °C)	44 (10)	6 (1)	12 (3)	0.13 (0.06–0.31)	<0.001	0.30 (0.15–0.57)	<0.001	2.27 (0.84–6.12)	0.11
Fever with unknown temperature	41 (10)	5 (1)	10 (3)	0.12 (0.05–0.30)	<0.001	0.26 (0.13–0.54)	<0.001	2.29 (0.77–6.80)	0.14
Any bodily pain	157 (36)	51 (13)	77 (21)	0.23 (0.16–0.33)	<0.001	0.45 (0.33–0.63)	<0.001	1.94 (1.31–2.88)	0.001
Headache	77 (18)	25 (6)	46 (13)	0.29 (0.18–0.47)	<0.001	NA		2.30 (1.37–3.88)	0.002
Myalgia	112 (26)	21 (5)	49 (13)	0.15 (0.09–0.24)	<0.001	0.44 (0.30–0.64)	<0.001	2.96 (1.73–5.07)	<0.001
Arthralgia	46 (11)	16 (4)	31 (8)	0.34 (0.19–0.61)	<0.001	0.77 (0.47–1.25)	0.28	2.26 (1.21–4.23)	0.01
Chest pain	12 (3)	4 (1)	3 (1)	NA		NA		NA	
Gastrointestinal reactions	106 (25)	25 (6)	52 (14)	0.20 (0.12–0.31)	<0.001	0.51 (0.35–0.73)	<0.001	2.57 (1.55–4.25)	<0.001
Poor appetite	78 (18)	12 (3)	36 (10)	0.14 (0.07–0.25)	<0.001	0.49 (0.32–0.76)	<0.001	3.64 (1.86–7.12)	<0.001
Nausea	39 (9)	10 (2)	17 (5)	0.25 (0.12–0.50)	<0.001	0.48 (0.27–0.88)	0.02	1.97 (0.88–4.38)	0.10
Vomiting	12 (3)	8 (2)	11 (3)	0.70 (0.28–1.73)	0.44	1.08 (0.47–2.49)	0.85	1.55 (0.62–3.92)	0.35
Diarrhea	21 (5)	3 (1)	12 (3)	0.14 (0.04–0.49)	<0.001	0.66 (0.32–1.36)	0.26	4.58 (1.28–16.44)	0.02
Skin reactions (noninjection site)	10 (2)	5 (1)	4 (1)	NA		NA		NA	
Rash	2 (0.5)	2 (0.5)	2 (0.5)	NA		NA		NA	
Redness (mucosa)	0 (0)	1 (0.2)	0 (0)	NA		NA		NA	
Itching	9 (2)	5 (1)	3 (1)	NA		NA		NA	
Other reactions	221 (51)	103 (25)	153 (42)	0.31 (0.23–0.42)	<0.001	0.68 (0.51–0.91)	0.01	2.20 (1.61–3.01)	<0.001
Chills	44 (10)	13 (3)	56 (15)	0.29 (0.15–0.54)	<0.001	1.59 (1.04–2.44)	0.03	5.55 (2.97–10.36)	<0.001
Fatigue	208 (48)	89 (22)	118 (32)	0.28 (0.21–0.39)	<0.001	0.50 (0.37–0.67)	<0.001	1.76 (1.27–2.45)	0.001
Sleepiness	116 (27)	56 (14)	76 (21)	0.43 (0.30–0.61)	<0.001	0.71 (0.51–0.99)	0.05	1.67 (1.14–2.45)	0.01
Shortness of breath	5 (1)	2 (0.5)	4 (1)	NA		NA		NA	
Chest tightness	14 (3)	4 (1)	9 (2)	0.30 (0.10–0.91)	0.03	0.75 (0.32–1.76)	0.51	2.54 (0.78–8.35)	0.12
Dizziness	58 (13)	20 (5)	23 (6)	NA		NA		NA	
Anaphylactic reaction	1 (0.2)	0 (0)	0 (0)	NA		NA		NA	
Daily activity was affected	19 (4)	6 (1)	10 (3)	0.32 (0.13–0.82)	0.02	0.60 (0.27–1.31)	0.20	1.86 (0.67–5.19)	0.24
Sought medical attention (e.g., visiting clinics)	4 (1)	2 (0.5)	7 (2)	NA				NA	
Required medications	118 (27)	30 (7)	67 (18)	0.19 (0.13–0.30)	<0.001	0.58 (0.41–0.83)	<0.001	3.00 (1.88–4.80)	<0.001
Acetaminophen	104 (24)	26 (6)	64 (17)	0.20 (0.12–0.32)	<0.001	0.66 (0.46–0.94)	0.02	3.31 (2.02–5.41)	<0.001
Required intravenous medication	7 (2)	1 (0.3)	2 (0.5)	NA		NA		NA	

Note. 99% of the participants received the ChAdOx1 vaccine as their first and second doses, and the mRNA-1273 vaccine accounted for 97% of their third doses.

Abbreviations. NA, not available due to nonconverged or unstable results.

Compared with the first dose, HD patients were less likely to report any adverse event (*p* < 0.001), any injection site pain (*p* < 0.001), any systemic reaction (*p* < 0.001), fever (*p* < 0.001), daily activity being affected (*p* = 0.02) and requiring medication (*p* < 0.001) after the second dose.

Compared with the first dose, HD patients were more likely to have any injection site reaction (*p* < 0.001) but were less likely to have any systemic reaction (*p* < 0.001), fever (*p* < 0.001) and requiring medication (*p* < 0.001) following the third dose.

Compared with the second dose, HD patients were more likely to report any adverse event (*p* < 0.001), any injection site reaction (*p* < 0.001), any systemic reaction (*p* < 0.001), fever (*p* < 0.001) and requiring medication (*p* < 0.001) after the third dose.

Table 3 describes the comparison of adverse events which resolved ≤2 days among the study participants who had the adverse events following COVID-19 vaccination. Most of the HD patients who experienced at least one injection site reaction had their reactions resolved within 2 days. The vast majority of HD patients with postvaccination fever experienced resolution within 2 days (95%, 100%, and 85% after the first, second, and third doses, respectively). More than 80 percent of HD patients who were taking medications to relieve postvaccination symptoms no longer required medications 2 days after vaccination.

**Table 3.** Comparison of adverse events which resolved ≤2 days among the study participants who had the adverse events following COVID-19 vaccination.

Type of reaction	First dose	Second dose	Third dose	Second vs. first		Third vs. first		Third vs. second	
	*n*/total (%)	*n*/total (%)	*n*/total (%)	Odds ratio (95% CI)	*p* value	Odds ratio (95% CI)	*p* value	Odds ratio (95% CI)	*p* value
Any injection site reaction	164/212 (77)	72/113 (64)	157/276 (57)	0.51 (0.31–0.84)	0.01	0.41 (0.27–0.61)	<0.001	0.80 (0.51–1.26)	0.34
Fever	125/131 (95)	17/17 (100)	39/46 (85)	NA		0.49 (0.12–2.01)	0.31	NA	
Any bodily pain	117/157 (75)	38/51 (75)	55/77 (71)	0.68 (0.31–1.51)	0.34	0.52 (0.27–1.00)	0.05	0.76 (0.33–1.73)	0.50
Gastrointestinal reactions	82/106 (77)	19/25 (76)	39/52 (75)	NA		NA		1.02 (0.08–13.66)	0.98
Skin reactions (noninjection site)	10/10 (100)	2/5 (40)	0/4 (0)	NA		NA		NA	
Other reactions	192/221 (87)	84/103 (82)	117/153 (76)	NA		NA		NA	
Required medications	107/118 (91)	26/30 (87)	59/67 (88)	NA		NA		NA	

Note. 99% of the participants received the ChAdOx1 vaccine as their first and second doses, and the mRNA-1273 vaccine accounted for 97% of their third doses.

Abbreviations. NA, not available due to nonconverged or unstable results.

### Factors associated with adverse events following three doses of COVID-19 vaccination

Table 4 presents the variables associated with the adverse events following three doses of the COVID-19 vaccination. After adjusting for significant variables in the GLMMs, older age (every 10-year increase of age) was found to be associated with a decreased risk of any adverse event (odds ratio [OR] 0.67, 95% confidence intervals [CI] 0.57–0.79), any injection site reaction (OR 0.66, 95% CI 0.57–0.77), and any systemic reaction (OR 0.66, 95% CI 0.57–0.76). Female sex was associated with an increased risk of any adverse event (OR 2.82, 95% CI 1.90–4.18), any injection site reaction (OR 2.04, 95% CI 1.43–2.91), and any systemic reaction (OR 2.32, 95% CI 1.64–3.30). Participants using an arteriovenous fistula for HD had an increased risk of any adverse event (OR 1.73, 95% CI 1.05–2.84) and any injection site reaction (OR 1.86, 95% CI 1.15 − 3.01), compared with those with an arteriovenous graft. Compared with ChAdOx1, mRNA-1273 was associated with an increased risk of any injection site reaction (OR 15.6, 95% CI 1.63–148.4).

**Table 4.** Variables associated with the adverse events following three doses of the COVID-19 vaccination.

	Any adverse event			Any injection site reaction			Any systemic reaction		
Variables	Odds ratio	95% CI	*p* value	Odds ratio	95% CI	*p* value	Odds ratio	95% CI	*p* value
Age (every increase of 10 years)	0.67	0.57 − 0.79	<0.001	0.66	0.57 − 0.77	<0.001	0.66	0.57 − 0.76	<0.001
Female sex (male as reference)	2.82	1.90 − 4.18	<0.001	2.04	1.43 − 2.91	<0.001	2.32	1.64 − 3.30	<0.001
AVF (AVG as reference)	1.73	1.05 − 2.84	0.03	1.86	1.15 − 3.01	0.01	1.33	0.84 − 2.11	0.23
mRNA-1273 (ChAdOx1 as reference)	10.5	0.77 − 144.6	0.08	15.6	1.63 − 148.4	0.02	4.86	0.79 − 29.86	0.09
Kt/V (Daugirdas) (every increase of 0.1)	0.96	0.88 − 1.05	0.40	1.02	0.94 − 1.11	0.64	0.92	0.85 − 1.00	0.05
BMI (every increase of 1 kg/m^2^)	1.00	0.96 − 1.05	0.89	1.02	0.98 − 1.07	0.31	0.99	0.95 − 1.03	0.57
Diabetes (no diabetes as reference)	1.15	0.81 − 1.63	0.44	0.75	0.54 − 1.05	0.09	1.21	0.88 − 1.67	0.24
Hypertension (no hypertension as reference)	1.11	0.79 − 1.57	0.55	1.10	0.80 − 1.52	0.56	1.12	0.82 − 1.53	0.48
Albumin (every increase of 1 g/dL)	0.80	0.45 − 1.40	0.43	0.78	0.46 − 1.31	0.34	0.75	0.45 − 1.25	0.27
Hemoglobin (every increase of 1 g/dL)	0.93	0.81 − 1.06	0.26	0.94	0.83 − 1.07	0.35	1.00	0.89 − 1.13	0.97
Ferritin (every increase of 100 ng/mL)	0.95	0.89 − 1.01	0.50	0.95	0.89 − 1.01	0.07	0.99	0.93 − 1.04	0.64
Intact PTH (every increase of 100 pg/dL)	0.99	0.95 − 1.03	0.08	0.97	0.93 − 1.01	0.11	1.01	0.98 − 1.05	0.46

Note. All of the above variables were adjusted in the multivariate analyses.

Abbreviations. AVF: arteriovenous fistula; AVG: arteriovenous graft; ChAdOx1: ChAdOx1 nCoV-19 [AstraZeneca]; mRNA-1273: mRNA-1273 SARS-CoV-2 [Moderna]: BMI: body mass index; CI: confidence intervals; DM: diabetes mellitus; HTN: hypertension; PTH: parathyroid hormone.

## Discussion

In this current study, a significant portion of the participants experienced at least one adverse event after each dose of the COVID vaccine. Most of the adverse events were mild, self-limited, and short-lived. A vast majority (99%) of the study participants received the ChAdOx1 vaccine as a homologous prime-boost regimen. Compared with the first dose, the second dose caused a significantly lower rate of adverse events. A substantial portion (97%) of the study participants received mRNA-1273 vaccine as their third dose and they experienced a higher rate of local injection site reactions and a lower rate of systemic reactions (except a higher rate of chills) compared with the first dose (99% received the ChAdOx1 vaccine). To compare the adverse events among three doses of COVID19 vaccination, several important clinical factors, including age, sex, body mass index, diabetes, hypertension, vaccine type, dialysis access type, Kt/V (Daugirdas), albumin, hemoglobin, ferritin, and intact parathyroid hormone, were included in the multivariate analyses using GLMMs. These models incorporated three repeated measurements of laboratory data and adverse event data arising from three doses of COVID-19 vaccination. We found that the risk of adverse events decreased with older age and increased with female sex and arteriovenous fistula. The HD patients who received mRNA-1273 vaccine experienced a higher rate of local injection site reaction compared with those with ChAdOx1 vaccine.

The commonly reported adverse events following three doses of COVID-19 vaccination in our study, including injection site reactions, fatigue, fever, myalgia, arthralgia, and headache, have also commonly been observed in other studies including non-dialysis populations [12–14]. In general, the adverse events noted in the current study resolved within 2 days after vaccination. This finding is in line with other studies showing that adverse events following COVID-19 vaccination are mostly short-lasting. Although the HD population is generally considered a frail group, following COVID-19 vaccination, few HD patients reported that their daily activity being affected and less than one-third of the participants required medication for the relief of symptoms, the majority were prescribed acetaminophen as the only treatment. Few patients sought medical attention in addition to receiving medical services from the dialysis unit. These findings implied that the COVID-19 vaccines were well tolerated among HD patients although they experienced a variety of adverse reactions. A recent study reported that patients receiving HD maintained some immune responses following natural COVID-19 infection [15]. Vaccination. with its well tolerance as observed in the current study, and valid immunogenicities as reported by previous studies, offers protection against COVID-19 without the risk of complications associated with natural COVID-19 infection.

In our study, a vast majority (99%) of the study participants received the ChAdOx1 vaccine as a homologous prime-boost regimen. We found that the reactogenicity of the second dose, including systemic reactions and local reactions, was lower than the first dose. This finding is compatible with previous studies surveying homologous prime-boost regimens in a non-dialysis population [16–18] that the second dose of ChAdOx1 elicited less adverse reactions, compared with the first dose. This is in contrast to reported profiles of mRNA vaccines where the reactogenicity increased with the second dose [19,20]. The reason for decreased reactogenicity of the boost dose of a homologous ChAdOx1 regime is not yet known. In the study conducted by Jordan R. Barrett et al. [16], they found that the antibodies against the ChAdOx1 vector were not further increased by the second dose and there was no association between reactogenicity and the presence or absence of antibodies to either SARS-CoV-2 or ChAdOx1 at the time of vaccination. Further research is required to ascertain the reason behind this phenomenon.

Since the majority of participants received a different type of vaccine as their third doses, the difference of adverse events between the third dose and the first two doses was probably due to the difference in types of vaccines but not the dosing of vaccines. To our knowledge, there was no study that compared postvaccination adverse events between vector-based vaccines and mRNA vaccines in the dialysis population. Our study found that the study participants experienced more injection site reactions but fewer systemic reactions following the third dose (97% received mRNA-1273) compared with the first dose (99% received ChAdOx1). In the multivariate analyses using GLMMs, mRNA-1273 vaccine was found to be associated with an increased risk of any injection site reaction compared with ChAdOx1 vaccine. These findings are in line with the study conducted by Cristina Menni et al. [12], which reported a higher rate of systemic side effects in those receiving ChAdOx1 than those with BNT162b2 vaccine, while the rate of local side effects was higher following BNT162b2 than ChAdOx1 vaccine. Thus, the findings of our study support the evidence that the dialysis population had a similar pattern of adverse events as the non-dialysis population, i.e., mRNA vaccine induced a higher rate of local injection site reactions but elicited a lower rate of systemic reactions, compared with ChAdOx1 vaccine.

In our study, older age was associated with a decreased risk of adverse events, while the female sex was noted to have an increased risk of adverse events, following COVID-19 vaccination. Younger age and female sex have been reported to be associated with a higher incidence of adverse events, following COVID-19 vaccination in the non-dialysis population [12,13,21,22]. In the study conducted by Cristina Menni et al. [12], following the first dose of either ChAdOx1or BNT162b2 vaccination, the proportion of participants who reported at least one systemic effect was significantly higher among people aged 55 years or younger than among those older than 55 years, and women were more likely to report adverse effects than men. In another study conducted by Alexis L. Beatty et al. [13] they found that younger age and female sex were among the strongest factors associated with adverse effects after BNT162b2 and mRNA-1273 vaccination. In a Japanese online survey study [21], adverse reactions were more frequently reported in females and younger individuals following vaccination with mRNA vaccines. Manfred S Green et al. [22] analyzed four cross-sectional studies about the adverse events following two to three doses of BNT162b2 vaccination and concluded that females of all ages had an increased risk of various postvaccination adverse events. However, in the study conducted by Gaetano Alfano et al. [23], they did not find an age-related difference in the rate of adverse events among HD patients who received two doses of mRNA-1273 vaccines. The differences in the effect of age and sex on the development of postvaccination adverse events may be due to the differences in vaccine types, number and dosing of vaccination, and study populations.

In the current study, we found that participants using an arteriovenous fistula for hemodialysis therapy were noted to have an increased risk of any adverse event, especially injection site reactions, compared with those with an arteriovenous graft. The mechanism behind the finding is not well known. It is plausible that the difference in clinical characteristics between patients with an arteriovenous fistula and those with an arteriovenous graft might explain the observed different rates in local reactogenicity. Vascular characteristics are one of the inherent differences between these two groups [24]. Patients with better vascular conditions are more likely to have an arteriovenous fistula created [25]. Since vascular response plays an important role in acute inflammation [26], it is reasonable that better peripheral circulation in patients with a functioning arteriovenous fistula may lead to more reactogenicity at the injection site following vaccination than those with an arteriovenous graft. Difference in comorbidities is another reason to explain why the risk of injection site reactions is higher in patients with an arteriovenous fistula than those with an arteriovenous graft. It has been shown that patients with an arteriovenous graft have a significantly higher number of comorbid conditions than those with an arteriovenous fistula [27]. A higher number of comorbidities is one of the factors associated with pain in patients on hemodialysis [28]. Taking the above factors into consideration, it is possible that patients with an arteriovenous graft who have more comorbidities might not report injection pain following vaccination as they have more preexisting body pain compared with those with an arteriovenous fistula. This issue may require further clarification in future studies.

## Limitations

A few limitations exist in this study. One is that the study participants were recruited from a single-center population; thus, the results of this study may not be generalizable to other populations. Second, the observation period was short, and we are therefore unable to provide information regarding the long-term adverse effects of the vaccines examined. Further prospective multicenter studies with longer durations of follow-up are warranted to provide more information regarding adverse events in this special population. Finally, we did not categorize the severity of adverse events. However, we observed that most of these adverse events were not severe, did not affect daily lifestyle activities, and could be managed with oral medication.

## Conclusions

Although the majority of the HD patients in our study experienced some adverse events following vaccination, the COVID-19 vaccines were generally well tolerated. Most of these adverse events were short-lived and easily handled. The vast majority (99%) of the participants received a homologous prime-boost regimen with ChAdOx1 and 97% received the mRNA-1273 as their third dose. Compared with the first dose, the second dose elicited less adverse events, while the third dose induced more injection site reactions but less systemic reactions. Age, sex, types of vascular access, and vaccines were associated with the risk of postvaccination adverse events. The findings in our study support the favorable safety profile of COVID-19 vaccination in HD patients and provide important clinical information regarding the risk assessment of postvaccination adverse events in this unique population.

## Data Availability

Individual-level deidentified participant data will be made available upon request by emailing the corresponding author. The data will be available for 3 years after publication.
